# Red meat and egg intake and serum ferritin concentrations in Colombian children: results of a population survey, ENSIN-2015

**DOI:** 10.1017/jns.2020.5

**Published:** 2020-03-25

**Authors:** Oscar F. Herran, Jhael N. Bermúdez, María Del Pilar Zea

**Affiliations:** 1 Universidad Industrial de Santander, Bucaramanga, Santander, Colombia; 2 Instituto Colombiano de Bienestar Familiar, Subdirección de Monitoreo y Evaluación, Bogotá, Colombia; 3 Facultad de Ciencias de la Salud, Universidad Javeriana de Cali, Cali, Colombia

**Keywords:** Nutritional surveys, Ferritin, Eggs, Meat, Diet, Colombia, ENSIN-2015, Encuesta Nacional de la Situación Nutricional en Colombia-2015 (National Survey of the Nutritional Situation of Colombia-2015), SF, serum ferritin

## Abstract

The present study aimed to (a) establish the frequency of consumption of red meat and eggs; (b) determine serum ferritin levels (μg/l); and (c) establish the relationship between serum ferritin and the consumption of red meat and eggs. In Colombia during 2014–2018, an analytical study was conducted in 13 243 Colombian children between the ages of 5 and 17 years, based on cross-sectional data compiled by ENSIN-2015 (Encuesta Nacional de la Situación Nutricional en Colombia-2015) on serum ferritin levels and dietary consumption based on a questionnaire of the frequency of consumption. Using simple and multiple linear regression, with the serum ferritin level as the dependent variable and the frequency of consumption as the main explanatory variable, the crude and adjusted partial regression coefficients (β) between serum ferritin levels and consumption were calculated. The frequency of habitual consumption of red meat was 0⋅49 (95 % CI 0⋅47, 0⋅51) times/d. The frequency of habitual egg consumption was 0⋅76 (95 % CI 0⋅74, 0⋅78) times per d. The mean serum ferritin level in men was 41⋅9 (95 % CI 40⋅6, 43⋅1) μg/l and in women, 35⋅7 (95 % CI 34⋅3, 37⋅7) μg/l (*P* < 0⋅0001). The adjusted β between the consumption of red meat and eggs and serum ferritin levels were β = 3⋅0 (95 % CI 1⋅2, 4⋅7) and β = 2⋅5 (95 % CI 1⋅0, 3⋅9) for red meat and eggs, respectively. In conclusion, red meat and eggs are determinants of serum ferritin levels in Colombia and, therefore, could be considered public policy options to reduce anaemia and Fe deficiency.

Serum ferritin (SF; μg/l) is a good indicator of body Fe deposits and is the most specific laboratory test to determine Fe deficiency^(1)^. A low level without other abnormal results means depletion of Fe deposits^(1)^, while elevated values (in adults) increase the risk of altered glucose homeostasis, insulin resistance syndrome and CVD in carriers of hereditary haemochromatosis^(2)^. However, the concentration of SF may be falsely high as a consequence of an inflammatory response in acute processes or tissue damage in chronic processes^(1–4)^. According to the Food and Nutrition Board of the United States National Academies of Sciences^(5)^, the dietary reference intakes of Fe range between 4⋅1 and 7⋅9 mg/d for the 4–17 years age group^(5)^. Fe present in animal-source foods is more bioavailable because it is found in its haem version, whose absorption is up to three times higher compared with non-haem Fe^(1,5)^. Red meat and eggs are part of the traditional dietary pattern of the Colombian population^(6–8)^, and the prevalence of consumption in a month for these two items is over 90 %^(9,10)^. In addition, they are sources of haem Fe: 100 g of red meat and eggs have 2⋅7 and 1⋅7 mg, respectively^(11)^, which is equivalent to 34 and 21 % of the recommended dietary intake/d for children between 5 and 17 years of age^(5)^.

According to the latest Encuesta Nacional de la Situación Nutricional en Colombia (National Survey of the Nutritional Situation of Colombia (ENSIN-2015)), Fe deficiency (SF<15 μg/l) in children between 5 and 12 years of age was 8⋅8 %, and Fe-deficiency anaemia (Hb<120 g/l) was 17⋅7 %. In children between 13 and 17 years, deficiency was 10⋅3 % and anaemia due to deficiency was 15⋅3 % (Hb<120 g/l in women and Hb<130 g/l in men), figures that are considered public nutrition issues^(10,12)^; this situation is common in developing countries^(13)^. Anaemia is the best-known consequence of all those resulting from Fe deficiency, but decreased muscle strength and ability to work^(14)^, along with altered immune system and inability to regulate body temperature, are also common^(15,16)^. These are the most relevant outcomes at an early age but, in the long term, behavioural alterations and irreparable cognitive deficits in children may be observed^(17–19)^.

Prevalence of usual consumption of red meat and eggs greater than 90 % confirm that the goal set for 2015 in the National Development Plan (Plan Nacional de Desarrollo (PND)) Colombia 2014–2018 was reached^(20)^, although the recommendation to increase the percentage of people who consume meat and eggs on a daily basis should technically be understood as increasing the frequency/d of consumption rather than the prevalence. Furthermore, the Food-Based Dietary Guidelines for the Colombian population over 2 years of age^(21)^ also make explicit recommendations on consumption: eating one egg, milk and dairy products daily. Although both documents recommend increasing the consumption of these foods, they do so from the perspective of sources of protein and not haem Fe.

The present study was proposed given that anaemia and Fe deficiency are endemic problems in the Colombian population and have devastating effects on children and on productivity in general. The objectives were: (a) to establish the frequency of red meat and egg consumption (times/d); (b) to determine SF levels among red meat and egg consumers; and (c) to establish the degree of relationship between SF and red meat and egg consumption in the population aged 5–17 years in Colombia.

## Materials and methods

The present analytical study is based on cross-sectional data collected in 2015–2016 by the ENSIN-2015 regarding SF levels (μg/l) and dietary consumption, using an FFQ.

### Study population

The ENSIN-2015^(9,10)^ was carried out during the past 4 years in Colombia. In short, these surveys are designed to represent 99 % of the population through stratified multistage sampling. All thirty-three geodemographic units or departments are grouped based on similar geographic and sociodemographic characteristics. The municipalities in these units are randomly selected and form strata with a probability proportional to the size of the population. Clusters of approximately ten households are randomly selected within these strata and household members are invited to participate. The ENSIN-2015 included 44 202 households, which represented 4739 clusters of 177 strata. Consent to participate was obtained during the field operation.

### Population and sample

The ENSIN-2015 included 151 343 people. The FFQ was applied to 28 903 subjects, of whom 14 092 were between 5 and 17 years old; they were inquired about their usual food intake in the last 30 d. SF levels were obtained in 41 439 subjects, of whom 36 978 were between 5 and 17 years old. A total of 28 253 subjects consumed two of the thirty-nine food items included in the FFQ, that is ‘Eggs’ and ‘Beef, veal, pork, capybara, rabbit, goat, domestic guinea pig’ – called red meat for the purposes of this study. The target population was redefined through subpopulations of interest. The first subpopulation was composed of children between 5 and 17 years old, consumers of red meat, who will not try any type of diet by medical prescription, without pregnancy, and with size ≥80 cm and size <200 cm and weight ≥12 kg and weight <200 kg in order to guarantee plausible data of nutritional status; this subpopulation included 12 106 children. The second subpopulation was defined as children between 5 and 17 years old, egg consumers, who will not try any type of diet by medical prescription, without pregnancy, and with size ≥80 cm and size <200 cm and weight ≥12 kg and weight <200 kg in order to guarantee plausible data of nutritional status; this subpopulation was made up of 12 702 children. Finally, a third subpopulation was established with the same characteristics described above, which allowed studying the children who usually consumed: (a) red meat and eggs; (b) red meat but not eggs; (c) no red meat but eggs; and (d) neither one; this subpopulation comprised 13 243 children. Since FFQ and SF data were obtained from different subsamples, 10 260 children had SF data in the red meat consumers group, 10 764 in the egg consumers group, and 16 582 in the red meat and egg consumers group.

### Data sources

Trained staff administered questionnaires to household heads to obtain information on sociodemographics, food security and level of wealth of the household. In addition, nutritionists applied in randomly selected subsamples an FFQ for the last month, with nine categories to establish the frequency of usual food intake. Children under the age of 12 years were assisted by their caregivers to respond to the FFQ. The food and food group checklist was designed by nutritionists based on the nutrition problems identified in past ENSIN^(9,10)^ and other sources. The list of items related to the response to frequency of consumption was adapted from two reproducibility and validity studies from other FFQ conducted in the Colombian population^(22,23)^. Face validity was guaranteed for all items on the checklist. The FFQ methodology is in nutritional epidemiology the most used in the world. The measurement with an FFQ allows estimating the prevalence of consumption and its frequency in terms of times/d. These two indicators of consumption are good estimators of the amount consumed (g). Establishing the amount (g) of consumption through other methods, such as the 24-h recall or diet record methods, is complex and very expensive, which makes it unfeasible^(22,23)^.

Anthropometric measurements were also taken by trained nutritionists and surveyors, using standardised techniques and calibrated equipment. Size was established using a portable measuring rod (Shorr Board Productions LCC), approaching the nearest 1 mm. Weight was established with SECA scales (model 874), approximating to 100 g. The nine responses in the FFQ on the frequency of consumption of these two food items were converted to a continuous variable, ‘times/d’.

SF levels were established in a random subsample of participants; for this purpose, blood was drawn by venepuncture from the median cubital vein. One sample was collected in an EDTA tube for plasma separation and another in a metal-free polypropylene tube without anticoagulant for serum separation. All samples were stored in liquid N_2_ until their processing at the National Health Institute (Instituto Nacional de Salud) of Colombia. Plasma ferritin was quantified in children aged 5 to 17 years using a competitive chemiluminescent immunoassay on an ADVIA Centaur analyser (Siemens HealthCare Diagnostics, Inc.). Plasma C-reactive protein (PCR) was quantified by turbidimetry in an ACS-180 analyser (Siemens Healthcare Diagnostics, Inc.). Since there is evidence of high SF values when there is acute or chronic inflammation^(1–4)^, and to ensure the plausibility of the results, the ENSIN-2015 corrected the SF values by the PCR level in children between 5 and 17 years old (SF = SF_original_ −15, if PCR>0⋅5).

The variables of interest were the frequency of usual consumption (times/d) of red meat, eggs and the SF level. The two FFQ items ‘Eggs’ and ‘Beef, veal, pork, capybara, rabbit, goat, domestic guinea pig’ were chosen because of their high consumption prevalence and because they are sources of haem Fe. Moreover, SF was preferred to Hb, which is a protein used as a biomarker of Fe stores and Fe nutrition. Other covariates were also considered to correlate them with dietary intake and SF levels: sex, age, size, BMI – and its equivalent according to the International Obesity Task Force (IOTF)^(24,25)^ –, state of food security in the household, wealth index, ethnicity, level of urbanism and the geographical region where the subjects live. The level of urbanism refers to people living in urban areas, including large cities. The rural category included suburban population centres close to small cities, municipal seats distant from small cities, and populations scattered or very distant from municipal seats. Household food security status was established using the Latin American and Caribbean Food Security (ELCSA) scale^(9)^.

On the other hand, wealth was established based on the index designed for the international Demographic and Health Survey^(26)^. This index was constructed through the analysis of main components based on household information, which included, among others, the type of construction material of the dwelling, the characteristics of the health services and, in general, the goods and services available in the household. The first component is used to create the index as a continuous variable (*z*-score), which is assigned to each subject within the household. The highest values represent the richest subjects. The wealth index was categorised by incorporating the complex sample design into quintiles according to the distribution achieved among all survey participants. The geographical region grouped several geodemographic units and, in general, they share the most relevant aspects of food culture^(27)^.

### Data processing and statistical analysis

All analyses were conducted using the analysis plan for complex sample designs of Stata software, version 14.1^(28)^. The analysis was conducted to estimate the average frequency of usual consumption (times/d) of red meat and eggs, with their corresponding 95 % CI, in the population of children aged 5 to 17 years and for each of the categories of covariates of interest. Using simple and multiple linear regression, having SF level as the dependent variable and consumption frequency as the main explanatory variable, crude and adjusted partial regression coefficients (β) were estimated – direction and strength of the association (correlation) – between the SF levels and the consumption frequency. In addition, adjusted differences between β were estimated for each of the covariate categories. To obtain the adjusted differences, a new term was created as the cross-product between frequency/d and SF level for each category of covariates (interaction). The adjusted differences and their corresponding 95 % CI incorporated the complex sample design and the multiple regression models included the following covariates: sex, age, stunted growth, BMI, food security level, wealth index, ethnicity, level of urbanism and region. Finally, using multiple linear regressions, crude and adjusted means and their corresponding 95 % CI were estimated for the SF levels in the consumption categories of the third subpopulation described above.

### Ethical considerations

All analyses were carried out under the principles of the Helsinki Declaration^(29)^. The used databases are in the public domain. This research is classified as ‘without risk’ according to Resolution 8430 of 1993 of the Colombian Ministry of Health^(30)^. Since this is a secondary analysis of population studies, with anonymised data, no authorisation is required from the Health Research Ethics Committee of the Industrial University of Santander.

## Results

**Fig. 1.**
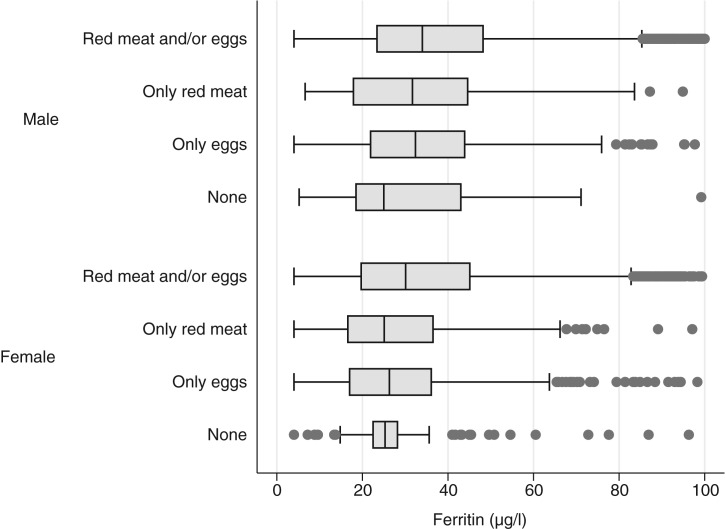
Serum ferritin levels in children between 5 and 17 years of age according to red meat and egg consumption, Colombia, ENSIN-2015 (Encuesta Nacional de la Situación Nutricional en Colombia-2015; National Survey of the Nutritional Situation of Colombia-2015). The line inside the box represents the median (quartile 2; Q2). The edges of the box represent quartile 1 (Q1) and quartile 3 (Q3), respectively. The limits of the whiskers represent up to 1·5 times the interquartile range (IQR = Q3 − Q1): Q1 − 1·5 or Q3 + 1·5. The points to the right and left represent extreme atypical values.

The prevalence of red meat consumption was 93⋅3 (95 % CI 92⋅4, 94⋅1) %, with no difference by sex (*P* = 0⋅809). The prevalence of egg consumption was 96⋅6 (95 % CI 96⋅0, 97⋅1) %, with no difference by sex (*P* = 0⋅174). The mean frequency of usual red meat consumption was 0⋅49 (95 % CI 0⋅47, 0⋅51) times/d, with no difference by sex (*P* = 0⋅764). The mean frequency of usual egg consumption was 0⋅76 (95 % CI 0⋅74, 0⋅78) times/d, with no difference by sex (*P* = 0⋅890). The mean SF was 38⋅9 (95 % CI 38⋅0, 39⋅9) μg/l; 41⋅9 (95 % CI 40⋅6, 43⋅1) μg/l in men and 35⋅7 (95 % CI 34⋅3, 37⋅7) μg/l in women. This difference of 6⋅2 μg/l against women is statistically significant (*P* < 0⋅0001). The proportion of children who ate red meat and/or eggs was 90⋅6 (95 % CI 89⋅5, 91⋅5) %, in the first category of the third subpopulation; more specifically, it was 2⋅7 (95 % CI 2⋅3, 3⋅2) % for children who only ate meat, 6⋅0 (95 % CI 5⋅3, 6⋅9) % for children that only ate eggs, and 0⋅6 (95 % CI 0⋅4, 1⋅0) % for children who do not eat meat or eggs. Among meat and/or egg consumers, the average SF was 39⋅4 (se 0⋅5) μg/l; among meat-only consumers 36⋅2 (se 2⋅0) μg/l; among egg-only consumers 34⋅5 (se 1⋅1) μg/l; and among those who do not eat meat nor eggs 34⋅2 (se 1⋅5) μg/l (*χ*^2^ for trend, *P* ≤ 0⋅0001) (Fig. 1).

The daily frequency of red meat consumption (times/d) was lower among those with stunted growth, as food insecurity increased, among black/Afro-descendant and indigenous populations, in rural areas and in all regions compared with the central region. SF (μg/l) levels among meat consumers were lower in women, as food insecurity increased, in the black/Afro-descendant population, and in all regions compared with the central region, except in the Eastern and Atlantic regions (Table 1). The frequency of egg consumption (times/d) was lower among overweight children and in all regions compared with the central region. Egg consumption was higher as the wealth index increased. SF levels among egg consumers were lower in women, in the black/Afro-descendant population and in the Atlantic, Pacific and Amazon/Orinoquia regions. Children who were overweight and obese had higher levels of SF than those classified as normal (Table 2).

**Table 1.** Red meat consumption (times/d) and serum ferritin levels (μg/l) among red meat consumers in a population between 5 and 17 years of age, Colombia, ENSIN-2015 (Mean values with their standard errors; differences and 95 % confidence intervals)

Variable	*n*	Red meat (times/d) (*n* 12 106)					Serum ferritin (μg/l) (*n* 10 260)				
		Mean	se	Difference	95 % CI	*P*	Mean	se	Difference	95 % CI	*P*
Total	12 106	0⋅49	0⋅01				39⋅2	0⋅50		38⋅2, 40⋅3	
Sex											
Male	6206	0⋅49	0⋅01	Ref		Ref	42⋅1	0⋅66	Ref		Ref
Female	5900	0⋅49	0⋅01	−0⋅00	−0⋅03, 0⋅02	0⋅764	35⋅9	0⋅63	−6⋅25	−7⋅87, −4⋅63	<0⋅0001
Age group (years)											
Children (5–10)	4176	0⋅48	0⋅01	Ref		Ref	39⋅4	0⋅76	Ref		Ref
Adolescents (11–17)	7930	0⋅50	0⋅01	0⋅02	−0⋅01, 0⋅05	0⋅149	39⋅1	0⋅61	−0⋅24	−2⋅10, 1⋅62	0⋅798
Age (years)											
5–8	2948	0⋅49	0⋅02	Ref		Ref	37⋅7	0⋅87	Ref		Ref
9–11	1903	0⋅46	0⋅02	−0⋅03	−0⋅07, 0⋅00	0⋅066	40⋅4	0⋅99	2⋅66	0⋅29, 5⋅01	0⋅028
12–15	4626	0⋅50	0⋅01	0⋅01	−0⋅03, 0⋅04	0⋅664	36⋅9	0⋅74	−0⋅80	−3⋅05, 1⋅45	0⋅479
16–17	2629	0⋅52	0⋅01	0⋅03	−0⋅01, 0⋅07	0⋅131	45⋅6	1⋅12	7⋅87	4⋅89, 10⋅85	<0⋅0001
Stunted growth (H/A)											
No	11 067	0⋅50	0⋅01	Ref		Ref	39⋅3	0⋅53	Ref		Ref
Yes (*z* < 2)	1035	0⋅44	0⋅02	−0⋅05	−0⋅10, −0⋅01	0⋅021	39⋅0	1⋅27	−0⋅27	−2⋅94, 2⋅40	0⋅839
Nutritional status (BMI)*											
No	10 110	0⋅49	0⋅01	Ref		Ref	38⋅4	0⋅54	Ref		Ref
Overweight (≥25 kg/m^2^)	1546	0⋅48	0⋅02	−0⋅01	−0⋅04, 0⋅03	0⋅753	42⋅7	1⋅17	4⋅24	1⋅79, 6⋅70	0⋅001
Obese (≥30 kg/m^2^)	450	0⋅55	0⋅04	0⋅10	−0⋅02, 0⋅14	0⋅131	45⋅2	2⋅63	6⋅75	1⋅40, 12⋅10	0⋅014
Food insecurity†											
No	3855	0⋅56	0⋅01	Ref		Ref	41⋅1	0⋅88	Ref		Ref
Mild	4355	0⋅50	0⋅01	−0⋅06	−0⋅10, −0⋅02	0⋅002	37⋅9	0⋅68	−3⋅23	−5⋅46, −1⋅01	0⋅005
Moderate	2260	0⋅44	0⋅02	−0⋅12	−0⋅17, −0⋅07	<0⋅0001	39⋅7	0⋅99	−1⋅43	−3⋅94, 1⋅08	0⋅259
Severe	1636	0⋅38	0⋅02	−0⋅18	−0⋅22, 0⋅14	<0⋅0001	37⋅8	1⋅35	−3⋅27	−6⋅46, −0⋅07	0⋅045
Wealth index											
Q_1_ – the poorest	2393	0⋅46	0⋅03	Ref		Ref	39⋅1	1⋅47	Ref		Ref
Q_2_	2548	0⋅48	0⋅02	0⋅01	−0⋅04, 0⋅07	0⋅568	37⋅8	0⋅92	−1⋅34	−4⋅63, 1⋅94	0⋅417
Q_3_	2570	0⋅46	0⋅02	−0⋅00	−0⋅07, 0⋅06	0⋅984	39⋅0	1⋅02	−0⋅18	−3⋅74, 3⋅38	0⋅919
Q_4_	2384	0⋅48	0⋅01	0⋅02	−0⋅04, 0⋅08	0⋅547	39⋅4	0⋅93	0⋅29	−3⋅10, 3⋅69	0⋅864
Q_5_ – the richest	2211	0⋅55	0⋅02	0⋅08	0⋅02, 0⋅15	0⋅017	40⋅3	1⋅00	1⋅17	−2⋅35, 4⋅70	0⋅508
Ethnicity											
Mestizo	9930	0⋅51	0⋅01	Ref		Ref	39⋅7	0⋅54	Ref		
Black/Afro-descendant	1061	0⋅40	0⋅04	−0⋅11	−0⋅18, −0⋅04	0⋅003	33⋅1	2⋅90	−6⋅52	−12⋅43, −0⋅62	0⋅031
Indigenous	1022	0⋅38	0⋅02	−0⋅13	−0⋅17, −0⋅08	<0⋅0001	38⋅6	1⋅44	−1⋅06	−4⋅12, 2⋅00	0⋅492
Area											
Urban	8896	0⋅48	0⋅01	0⋅03	−0⋅01, 0⋅07	0⋅187	39⋅3	0⋅55	Ref		Ref
Rural	3210	0⋅51	0⋅02	0⋅48	0⋅46, 0⋅50	<0⋅0001	39⋅2	1⋅03	−0⋅09	−2⋅35, 2⋅17	0⋅937
Region											
Central	2961	0⋅63	0⋅03	Ref			39⋅9	0⋅82	Ref		Ref
Atlantic	2242	0⋅38	0⋅01	−0⋅25	−0⋅31, −0⋅19	<0⋅0001	35⋅4	1⋅09	−4⋅52	−7⋅25, −1⋅79	0⋅002
Eastern	2134	0⋅55	0⋅02	−0⋅08	−0⋅15, −0⋅01	0⋅032	43⋅8	1⋅27	3⋅92	0⋅90, 6⋅94	0⋅012
Pacific	1490	0⋅43	0⋅02	−0⋅20	−0⋅27, −0⋅13	<0⋅001	35⋅7	0⋅97	−4⋅17	−6⋅71, −1⋅62	0⋅002
Bogotá	833	0⋅47	0⋅02	−0⋅15	−0⋅22, −0⋅08	<0⋅001	45⋅1	1⋅41	5⋅25	1⋅98, 8⋅52	0⋅002
Amazon/Orinoquia	2446	0⋅46	0⋅02	−0⋅17	−0⋅24, −0⋅10	<0⋅001	35⋅8	1⋅40	−4⋅06	−7⋅31, −0⋅81	0⋅015

ENSIN-2015, Encuesta Nacional de la Situación Nutricional en Colombia-2015 (National Survey of the Nutritional Situation of Colombia-2015); Ref, reference; H/A, height for age; Q, quintile.

* Based on BMI equivalents according to the International Obesity Task Force.

† Based on the Latin American and Caribbean Food Security (ELCSA) Scale.

**Table 2.** Egg consumption (times/d) and serum ferritin levels (μg/l) among egg consumers in a population between 5 and 17 years of age, Colombia, ENSIN-2015 (Mean values with their standard errors; differences and 95 % confidence intervals)

Variable	*n*	Eggs (times/d) (*n* 12 702)					Serum ferritin (μg/l) (*n* 10 764)				
		Mean	se	Difference	95 % CI	*P*	Mean	se	Difference	95 % CI	*P*
Total	12 702	0⋅76	0⋅01				39⋅0	0⋅49			
Sex											
Male	6521	0⋅76	0⋅01	Ref			42⋅0	0⋅64	Ref		Ref
Female	6181	0⋅75	0⋅01	−0⋅00	−0⋅04, 0⋅03	0⋅890	35⋅6	0⋅61	−6⋅38	−7⋅97, −4⋅78	<0⋅001
Age group (years)											
Children (5–10)	4485	0⋅75	0⋅01	Ref		Ref	39⋅4	0⋅76	Ref		Ref
Adolescents (11–17)	8217	0⋅76	0⋅01	0⋅01	−0⋅02, 0⋅05	0⋅485	39⋅1	0⋅61	−0⋅39	−2⋅20, 1⋅42	0⋅665
Age (years)											
5–8	3171	0⋅76	0⋅01	Ref		Ref	37⋅7	0⋅87	Ref		Ref
9–11	2036	0⋅74	0⋅02	−0⋅02	−0⋅07, 0⋅02	0⋅292	40⋅4	0⋅99	2⋅46	0⋅21, 4⋅71	0⋅033
12–15	4827	0⋅76	0⋅02	−0⋅00	−0⋅04, 0⋅04	0⋅930	36⋅9	0⋅74	−0⋅64	−2⋅84, 1⋅56	0⋅564
16–17	2668	0⋅78	0⋅02	0⋅02	−0⋅02, 0⋅07	0⋅323	45⋅6	1⋅12	7⋅64	4⋅73, 10⋅54	<0⋅0001
Stunted growth (H/A)											
No	11 557	0⋅75	0⋅01	Ref		Ref	39⋅3	0⋅53	Ref		Ref
Yes (*z* < 2)	1140	0⋅74	0⋅04	−0⋅01	−0⋅10, 0⋅07	0⋅748	39⋅0	1⋅27	−1⋅03	−3⋅53, 1⋅4	0⋅412
Nutritional status (BMI)*											
No	10 611	0⋅76	0⋅01	Ref		Ref	38⋅4	0⋅54	Ref		Ref
Overweight (≥25 kg/m^2^)	1629	0⋅71	0⋅02	−0⋅05	−0⋅09, −0⋅00	0⋅034	42⋅7	1⋅17	4⋅04	1⋅66, 6⋅44	0⋅001
Obese (≥30 kg/m^2^)	462	0⋅81	0⋅04	0⋅06	−0⋅03, 0⋅14	0⋅215	45⋅2	2⋅63	6⋅96	1⋅49, 12⋅42	0⋅013
Food insecurity†											
No	3973	0⋅76	0⋅02	Ref		Ref	41⋅1	0⋅88	Ref		Ref
Mild	4513	0⋅78	0⋅01	0⋅02	−0⋅02, 0⋅06	0⋅352	37⋅9	0⋅68	−2⋅80	−4⋅97, −0⋅62	0⋅012
Moderate	2409	0⋅75	0⋅02	−0⋅02	−0⋅07, 0⋅04	0⋅504	39⋅7	0⋅99	−1⋅84	−4⋅26, 0⋅58	0⋅134
Severe	1807	0⋅68	0⋅03	−0⋅08	−0⋅14, −0⋅02	0⋅015	37⋅8	1⋅35	−3⋅53	−6⋅49, −0⋅56	0⋅020
Wealth index											
Q_1_ – the poorest	2639	0⋅66	0⋅03	Ref		Ref	39⋅1	1⋅47	Ref		Ref
Q_2_	2693	0⋅69	0⋅02	0⋅03	−0⋅05, 0⋅10	0⋅465	37⋅8	0⋅92	−1⋅85	−4⋅89, 1⋅19	0⋅228
Q_3_	2665	0⋅77	0⋅02	0⋅11	0⋅03, 0⋅19	0⋅011	39⋅0	1⋅02	−0⋅15	−3⋅51, 3⋅20	0⋅926
Q_4_	2461	0⋅79	0⋅02	0⋅12	0⋅05, 0⋅20	0⋅001	39⋅4	0⋅93	0⋅21	−2⋅94, 3⋅35	0⋅897
Q_5_ – the richest	2244	0⋅81	0⋅02	0⋅15	0⋅07, 0⋅23	<0⋅0001	40⋅3	1⋅00	1⋅16	−2⋅16, 4⋅47	0⋅489
Ethnicity											
Mestizo	10 268	0⋅76	0⋅01	Ref		Ref	39⋅7	0⋅54	Ref		Ref
Black/Afro-descendant	1269	0⋅68	0⋅08	−0⋅08	−0⋅25, 0⋅09	0⋅335	33⋅1	2⋅90	−6⋅36	−11⋅6, −1⋅07	0⋅019
Indigenous	1067	0⋅75	0⋅03	−0⋅01	−0⋅08, 0⋅05	0⋅662	38⋅6	1⋅44	−1⋅57	−4⋅39, 1⋅24	0⋅268
Area											
Urban	9346	0⋅77	0⋅01	Ref		Ref	39⋅3	0⋅55	Ref		
Rural	3356	0⋅72	0⋅02	−0⋅05	−0⋅10, 0⋅00	0⋅058	39⋅2	1⋅03	−0⋅02	−2⋅18, 2⋅13	
Region											
Central	3104	0⋅86	0⋅02	Ref		Ref	39⋅9	0⋅82	Ref		Ref
Atlantic	2250	0⋅57	0⋅02	−0⋅29	0⋅34, −0⋅24	<0⋅0001	35⋅4	1⋅09	−3⋅88	−6⋅52, −1⋅24	0⋅005
Eastern	2208	0⋅82	0⋅02	−0⋅03	−0⋅09, 0⋅02	0⋅248	43⋅8	1⋅27	4⋅00	1⋅01, 6⋅99	0⋅010
Pacific	1592	0⋅78	0⋅03	−0⋅08	−0⋅16, −0⋅07	0⋅032	35⋅7	0⋅97	−3⋅92	−6⋅36, −1⋅48	0⋅002
Bogotá	853	0⋅79	0⋅02	−0⋅07	−0⋅12, −0⋅02	0⋅013	45⋅1	1⋅41	6⋅27	3⋅00, 9⋅53	<0⋅0001
Amazon/Orinoquia	2695	0⋅78	0⋅02	−0⋅07	−0⋅13, −0⋅02	0⋅009	35⋅8	1⋅40	−3⋅50	−6⋅65, −0⋅34	0⋅031

ENSIN-2015, Encuesta Nacional de la Situación Nutricional en Colombia-2015 (National Survey of the Nutritional Situation of Colombia-2015); Ref, reference; H/A, height for age; Q, quintile.

* Based on BMI equivalents according to the International Obesity Task Force.

† Based on the Latin American and Caribbean Food Security (ELCSA) Scale.

**Fig. 2.**
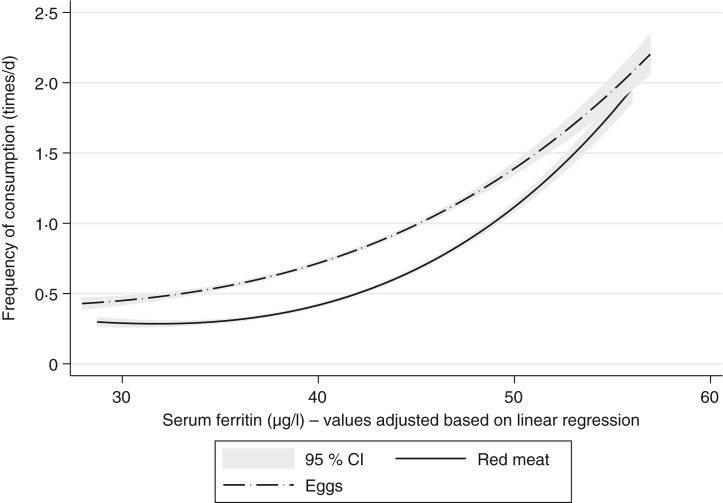
Adjusted serum ferritin levels and frequency of red meat and egg consumption (times/d). Colombia, ENSIN-2015 (Encuesta Nacional de la Situación Nutricional en Colombia-2015; National Survey of the Nutritional Situation of Colombia-2015).

Tables 3 and 4 present the raw and adjusted β-coefficients between red meat and egg consumption (times/d) and SF levels (μg/l). The correlation between red meat and egg consumption and SF levels is evident: β = 3⋅0 (95 % CI 1⋅2, 4⋅7) and β = 2⋅5 (95 % CI 1⋅0, 3⋅9) for red meat and eggs, respectively. There were no differences in the correlations adjusted by the categories of the variables studied, except for Bogotá, where the correlation between egg consumption and SF levels was 5⋅4 times higher than the mean correlation in the general population (β = 13⋅4 (95 % CI 5⋅5, 21⋅3); Table 4). Fig. 2 shows how SF levels increase as the frequency of meat and eggs consumption increases.

**Table 3.** Partial regression coefficients (β), crude and adjusted, between serum ferritin (γ) and red meat consumption (*Xi*) (times/d) in a population between 5 and 17 years of age, Colombia, ENSIN-2015

Variable	Crude			Adjusted difference		
	β*	95 % CI	*P*	β†	95 % CI	*P*
Total	3⋅26	1⋅46, 5⋅07	0⋅001	2⋅99	1⋅18, 4⋅73	
Sex						
Male	4⋅52	1⋅90, 7⋅14	0⋅001	Ref	Ref	Ref
Female	1⋅92	−0⋅41, 4⋅24	0⋅104	−2⋅57	−5⋅92, 0⋅78	0⋅131
Age group (years)						
Children (5–10)	3⋅05	0⋅05, 6⋅04	0⋅046	Ref	Ref	Ref
Adolescents (11–17)	3⋅44	1⋅03, 5⋅85	0⋅006	−0⋅23	−4⋅11, 3⋅67	0⋅908
Age (years)						
5–8	3⋅74	0⋅60, 6⋅89	0⋅020	1⋅73	−2⋅63, 6⋅10	0⋅430
9–11	2⋅15	−1⋅54, 5⋅83	0⋅248	Ref	Ref	Ref
12–15	3⋅89	0⋅44, 7⋅34	0⋅028	1⋅26	−3⋅85, 6⋅37	0⋅624
16–17	2⋅53	−1⋅83, 6⋅88	0⋅250	−0⋅50	−6⋅01, 5⋅92	0⋅987
Stunted growth						
No	3⋅36	1⋅48, 5⋅23	0⋅001	Ref	Ref	Ref
Yes (*z* < 2)	2⋅07	−3⋅22, 7⋅37	0⋅436	−1⋅44	−7⋅24, 4⋅35	0⋅620
Nutritional status (BMI)‡						
No	2⋅71	0⋅75, 4⋅66	0⋅008	−2⋅80	−9⋅41, 3⋅80	0⋅399
Overweight (≥25 kg/m^2^)	4⋅94	−0⋅95, 10⋅83	0⋅098	Ref	Ref	Ref
Obese (≥30 kg/m^2^)	6⋅21	−2⋅22, 14⋅65	0⋅133	0⋅96	−9⋅78, 11⋅69	0⋅859
Food insecurity§						
No	3⋅88	1⋅01, 6⋅74	0⋅009	0⋅58	−5⋅83, 6⋅99	0⋅857
Mild	1⋅75	−1⋅38, 4⋅89	0⋅267	−1⋅64	−7⋅65, 4⋅36	0⋅586
Moderate	4⋅20	−1⋅07, 9⋅47	0⋅116	Ref	Ref	Ref
Severe	3⋅10	−1⋅33, 7⋅52	0⋅165	−1⋅84	−8⋅99, 5⋅31	0⋅609
Wealth index						
Q_1_ – the poorest	5⋅95	2⋅05, 9⋅84	0⋅004	4⋅59	−1⋅71, 10⋅89	0⋅151
Q_2_	2⋅98	0⋅16, 5⋅80	0⋅039	1⋅77	−3⋅75, 7⋅29	0⋅524
Q_3_	4⋅91	1⋅37, 8⋅44	0⋅008	3⋅39	−2⋅12, 8⋅90	0⋅223
Q_4_	1⋅19	−3⋅22, 5⋅60	0⋅588	Ref	Ref	Ref
Q_5_ – the richest	1⋅04	−3⋅65, 5⋅72	0⋅651	−0⋅25	−7⋅04, 6⋅54	0⋅942
Ethnicity						
Mestizo	3⋅06	1⋅10, 5⋅02	0⋅003	−0⋅19	−6⋅73, 6⋅35	0⋅953
Black/Afro-descendant	4⋅23	−2⋅06, 10⋅53	0⋅170	−0⋅11	−9⋅05, 8⋅82	0⋅980
Indigenous	3⋅34	−3⋅89, 10⋅57	0⋅334	Ref	Ref	Ref
Area						
Urban	2⋅19	−0⋅27, 4⋅65	0⋅080	Ref	Ref	Ref
Rural	4⋅89	2⋅11, 7⋅67	0⋅001	3⋅23	−0⋅53, 6⋅99	0⋅091
Region						
Central	4⋅50	2⋅10, 6⋅89	0⋅001	1⋅34	−4⋅88, 7⋅56	0⋅669
Atlantic	1⋅56	−4⋅05, 7⋅17	0⋅558	−1⋅25	−9⋅00, 6⋅51	0⋅749
Eastern	2⋅84	−1⋅87, 7⋅56	0⋅218	−1⋅33	−8⋅50, 5⋅84	0⋅712
Pacific	−0⋅29	−4⋅81, 4⋅24	0⋅892	−3⋅41	−10⋅57, 3⋅74	0⋅344
Bogotá	−3⋅38	Nd	Nd	−6⋅45	−17⋅64, 4⋅74	0⋅253
Amazon	3⋅10	−4⋅38, 10⋅58	0⋅360	Ref	Ref	Ref

ENSIN-2015, Encuesta Nacional de la Situación Nutricional en Colombia-2015 (National Survey of the Nutritional Situation of Colombia-2015); Ref, reference; Q, quintile; Nd, no data.

* Partial regression coefficient achieved in a simple linear regression model.

† Partial regression coefficient achieved in a multiple linear regression model, where the dependent variable is the serum ferritin level (μ/l), and the main explanatory variable is the frequency/d of red meat consumption. The model was adjusted for sex, age, nutritional status, level of food insecurity in the household, wealth index, ethnicity, and geographical area and region.

‡ Based on BMI equivalents according to the International Obesity Task Force.

§ Based on the Latin American and Caribbean Food Security (ELCSA) Scale.

**Table 4.** Partial regression coefficients (β), crude and adjusted, between serum ferritin *(*γ) and egg consumption (*Xi*) (times/d) in a population between 5 and 17 years of age, Colombia, ENSIN-2015

	Crude			Adjusted difference†		
Variable	β*	95 % CI	*P*	β	95 % CI	*P*
Total	2⋅66	1⋅10, 4⋅21		2⋅47	1⋅01, 3⋅94	
Sex						
Male	3⋅35	1⋅08, 5⋅62	0⋅004	Ref	Ref	Ref
Female	1⋅92	0⋅16, 3⋅68	0⋅003	−1⋅16	−3⋅94, 1⋅62	0⋅407
Age group (years)						
Children (5–10)	2⋅82	0⋅61, 5⋅04	0⋅013	Ref	Ref	Ref
Adolescents (11–17)	2⋅55	0⋅40, 4⋅69	0⋅021	0⋅02	−3⋅00, 3⋅05	0⋅989
Age (years)						
5–8	2⋅95	0⋅45, 5⋅44	0⋅022	Ref	Ref	Ref
9–11	2⋅82	−0⋅15, 5⋅80	0⋅062	−0⋅31	−4⋅08, 3⋅45	0⋅869
12–15	4⋅16	0⋅99, 7⋅32	0⋅011	0⋅97	−2⋅95, 4⋅89	0⋅622
16–17	−1⋅12	−4⋅10, 1⋅85	0⋅453	−3⋅55	−7⋅31, 0⋅21	0⋅064
Stunted growth						
No	2⋅91	1⋅34, 4⋅49	<0⋅0001	Ref	Ref	Ref
Yes (*z* < 2)	−0⋅31	−4⋅87, 4⋅25	0⋅891	−3⋅78	−8⋅02, 0⋅46	0⋅080
Nutritional status (BMI)‡						
No	3⋅19	1⋅53, 4⋅85	<0⋅0001	3⋅85	−4⋅79, 12⋅49	0⋅376
Overweight (≥25 mg/m^2^)	−0⋅64	−5⋅28, 3⋅99	0⋅782	0⋅34	−9⋅46, 10⋅14	0⋅945
Obese (≥30 kg/m^2^)	−0⋅92	−10⋅28, 8⋅44	0⋅837	Ref	Ref	Ref
Food insecurity§						
No	3⋅27	0⋅04, 6⋅51	0⋅047	2⋅93	−1⋅90, 7⋅76	0⋅230
Mild	1⋅85	−0⋅32, 4⋅02	0⋅094	2⋅16	−2⋅24, 6⋅56	0⋅331
Moderate	4⋅92	1⋅63, 8⋅21	0⋅004	4⋅62	−0⋅30, 9⋅55	0⋅065
Severe	0⋅12	−3⋅82, 4⋅06	0⋅952	Ref	Ref	Ref
Wealth index						
Q_1_ – the poorest	1⋅81	−2⋅21, 5⋅84	0⋅370	−0⋅69	−5⋅50, 4⋅11	0⋅774
Q_2_	0⋅69	−2⋅17, 3⋅54	0⋅632	−1⋅80	−5⋅95, 2⋅36	0⋅391
Q_3_	2⋅56	−0⋅37, 5⋅50	0⋅085	Ref	Ref	Ref
Q_4_	2⋅03	−0⋅77, 4⋅82	0⋅150	−0⋅19	−4⋅07, 3⋅68	0⋅921
Q_5_ – the richest	4⋅77	0⋅74, 8⋅79	0⋅022	2⋅27	−2⋅53, 7⋅06	0⋅348
Ethnicity						
Mestizo	2⋅87	1⋅27, 4⋅47	0⋅001	1⋅12	−3⋅28, 5⋅54	0⋅611
Black/Afro-descendant	−2⋅04	−7⋅38, 3⋅31	0⋅429	−3⋅94	−10⋅65, 2⋅78	0⋅246
Indigenous	1⋅45	−2⋅95, 5⋅84	0⋅487	Ref	Ref	Ref
Area						
Urban	3⋅22	1⋅39, 5⋅06	0⋅001	Ref	Ref	Ref
Rural	1⋅27	−1⋅54, 4⋅09	0⋅369	−1⋅71	−4⋅98, 1⋅56	0⋅301
Region						
Central	1⋅25	−0⋅85, 3⋅35	0⋅222	2⋅38	−0⋅89, 5⋅64	0⋅150
Atlantic	0⋅07	−3⋅94, 4⋅08	0⋅970	0⋅65	−3⋅89, 5⋅20	0⋅774
Eastern	1⋅55	−1⋅91, 5⋅01	0⋅354	2⋅42	−1⋅53, 6⋅38	0⋅226
Pacific	0⋅63	−3⋅24, 4⋅51	0⋅727	1⋅99	−2⋅37, 6⋅35	0⋅365
Bogotá	12⋅88	Nd	Nd	13⋅40	5⋅51, 21⋅30	0⋅001
Amazon	−0⋅59	−3⋅95, 2⋅78	0⋅693	Ref	Ref	Ref

ENSIN-2015, Encuesta Nacional de la Situación Nutricional en Colombia-2015 (National Survey of the Nutritional Situation of Colombia-2015); Ref, reference; Q, quintile; Nd, no data.

* Partial regression coefficient achieved in a simple linear regression model.

† Partial regression coefficient achieved in a multiple linear regression model, where the dependent variable is the serum ferritin level (μ/l), and the main explanatory variable is the frequency/d of egg consumption. The model was adjusted for sex, age, nutritional status, level of food insecurity in the household, wealth index, ethnicity, and geographical area and region.

‡ Based on BMI equivalents according to the International Obesity Task Force.

§ Based on the Latin American and Caribbean Food Security (ELCSA) Scale.

## Discussion

It could be established that, in children between 5 and 17 years of age in Colombia, the average frequency (times/d) of usual consumption of red meat is lower than the consumption of eggs. Red meat is consumed every 2 d, while two eggs are consumed every 3 d. In addition, the prevalence of red meat and egg consumption is high, above 95 %, and combined is greater than 90 %. However, in order to meet the consumption/d targets established in the National Development Plan (Plan Nacional de Desarrollo (PND))^(20)^ and the Food-Based Dietary Guidelines^(21)^, the average frequency of red meat consumption among the population should be doubled – by 100 % – and egg consumption should be increased by at least 25 %. SF levels are very similar among meat and egg consumers; however, meat and/or egg consumers, who are the majority, have higher SF levels than consumers of one of the two foods exclusively (Fig. 1). The correlation between frequency of red meat and egg consumption is similar and statistically significant, which corroborated that these foods are determinants of SF levels and are, therefore, public policy options to decrease anaemia and Fe deficiency.

The mean frequency of red meat consumption was lower in children with stunted growth (chronic malnutrition), and inversely correlated to the levels of food insecurity in the household, in the indigenous and black/Afro-descendant ethnic groups, in the rural area and in all regions when compared with the central region. This finding may be explained to a large extent by the monetary poverty associated with undernourished individuals or ethnic minorities and, also, with a lower structural development in the peripheral regions or their proximal variables such as the rural area^(31–37)^. Having meat on the table requires a greater relative cost than that needed to have eggs, besides physical infrastructure to ensure the cold chain and others required for slaughter, which makes meat production more complex and costly. More precisely and based on red meat and egg prices in November 2018, 1 g of Fe derived from meat costs at least 1⋅5 times more than one egg derivative.

Red meat, according to the WHO through the International Agency for Research on Cancer (IARC)^(38,39)^, was classified as a group 2A agent: probable carcinogen. The dose (portion + frequency/d) of red meat consumed in Colombia by children between 5 and 17 years of age, according to the ENSIN-2005, was 54 g – average estimate derived from the current intake through the 24-h dietary recall^(9)^ –, and the frequency/d according to the ENSIN is decreasing: 0⋅57 in 2010 and 0⋅49 in 2015. At these doses, the risk for developing colorectal or other cancers is very low^(38–40)^; red meat is a risk factor for the development of cancer when the dose reaches average amounts greater than 100 g/d and a frequency of one or more times per d^(39–43)^. According to the Colombian Cattle Ranching Association (Federación Colombiana de Ganaderos (Fedegan)), in 2015, the consumption of beef among the general population was 19⋅1 kg per capita, while the consumption of pork was 7⋅8 kg per capita. The amount of beef, as well as its frequency, has been decreasing (it was 18⋅6 kg in 2017) but pork consumption is increasing (9⋅4 kg per capita)^(44)^. With this in mind, policies that tend to increase red meat consumption are useful to increase SF levels and do not generate cancer risk.

Due to their amino-acid content and bioavailability, eggs are among the best sources of protein^(39)^. In the 1990s, and even at the beginning of the 21st century, their consumption was questioned for their cholesterol content – between 300 and 400 mg per 100 g – even though there is not enough evidence to state that dietary cholesterol is a risk factor for the development of CVD^(45–48)^. According to the National Poultry Farmers Fund of Colombia (Fondo Nacional de Avicultores de Colombia) and the National Poultry Fund (Fondo Nacional Avícola (FENAVI)), the egg industry has been growing steadily in the last 9 years. In 2017, a figure of 279 units per capita consumed by the general population was reported and an increase of fifteen units was expected for 2018^(49)^. According to the ENSIN, the frequency/d in this age group also increased, going from 0⋅69 in 2010 to 0⋅76 in 2015.

In Colombia, as in many parts of the world, anaemia and Fe deficiency have been treated, among other interventions, with the fortification of wheat flour and other grains. According to Decree 1944 of October 28, 1996, wheat flour must be fortified with vitamin B_1_, vitamin B_2_, niacin, folic acid and Fe. Each kg of wheat flour should have at least 44 mg of Fe through ferrous fumarate^(50)^. In Colombia, only wheat flour is enriched. The consumption of eggs and red meat is visible, that of wheat flour is invisible. Flour is found in preparations such as bread, cakes, sauces, etc., which makes it difficult to estimate the prevalence, frequency/d and the amount consumed. Also, in Colombia, other situations make measurement even more difficult, e.g. there is the smuggling of flour at the borders; so much of what is consumed is not enriched. Enriched flour does not necessarily contain the same amounts, since the standard establishes a minimum level but is not mandatory, so some producers add ferrous fumarate above the minimum level. Therefore, it is not possible to introduce a non-haem indicator in the analysis.

Although many countries in the world fortify grains with Fe, few evaluate the impact of this intervention; Colombia is one of them. However, evidence consistently suggests that fortification increases SF levels, but not Hb levels^(51)^. The prevalence of anaemia and Fe deficiency in the Colombian population and in the age group studied here suggests that, while fortification of wheat flour is important as a public health measure, it is not sufficient, nor is it the only one that should be considered. Since the percentage of bioavailability of haem Fe is three times higher than that of non-haem Fe and, once consumed, its absorption can be increased up to four times if it is accompanied by acidic juices^(52)^, dietary interventions based on the consumption of red meat and eggs within a comprehensive strategy consistent with Food-Based Dietary Guidelines, which address other public health problems such as childhood obesity, should be considered^(21)^. Energy expenditure should be decisive in these strategies, because there is evidence of excessive protein consumption in children in Colombia^(6)^. In addition, red meat and eggs are part of the traditional consumption pattern, which actually protects children from excess weight in Colombia^(8)^.

In diets rich in cereals of vegetarians, the addition of eggs showed that due to phosphovitin contained in the yolk, Fe absorption may be decreased between 16 and 50 %^(53)^. However, in Colombia, eggs are not only consumed for Fe, but they are also the main source of protein of high biological value and the most economical. The prevalence of vegetarianism in Colombia is less than 1 %^(10)^, and in any case, these findings *in vitro* would be in favour of the association reported here, because if this effect were important in Colombia, it would mitigate the associations reported.

Finally, all structural aspects that directly or indirectly constitute barriers for red meat and eggs to reach the plates of children and the Colombian population, in general, or, specifically, to the detriment of their availability and consumption, both in quantity and frequency/d, are against public health. Discouraging rural development, taxing the basic food basket and foods that are traditionally consumed by the population, the armed conflict, disinformation about the dietary properties of food and their relationship with chronic disease, the inability to control borders and allow contraband, poor phytosanitary control in the regions, the lack of energy infrastructure and other reasons external to the nutrition sector are the structural aspects referred to.

### Scope and limitations of the study

Usual food consumption is stable in the medium and long term. The estimates made by the ENSIN-2005, and more recently by Federación Colombiana de Ganaderos (Fedegan) and Fondo Nacional Avícola (FENAVI), allow approximating reliably the amount (g) consumed by children. The frequency (times/d) estimated with an FFQ is the best mechanism to calculate the usual intake; therefore, the correlation found between SF levels and red meat and egg consumption with ENSIN-2015 data is plausible and also valid. The ENSIN-2015 is still being developed; future results of the 24-h recall, which allow incorporating the amount (g) of red meat and eggs consumed, will facilitate the understanding of the correlation between red meat and egg consumption with SF levels and the design of population-based dietary interventions. The results presented here on frequency/d of consumption can be used as a baseline to evaluate the impact of policies aimed at increasing red meat and egg consumption.
